# Observations of Nocturnal Sulfuric Acid Formation in Pittsburgh, PA

**DOI:** 10.1021/acs.est.5c14137

**Published:** 2026-04-10

**Authors:** Dominic A. Casalnuovo, Darren Cheng, Christine Troller, Ziheng Zeng, Albert A. Presto, Coty N. Jen

**Affiliations:** † Department of Chemical Engineering, 6612 Carnegie Mellon University, Pittsburgh, Pennsylvania 15213, United States; ‡ Center for Atmospheric Particle Studies, Carnegie Mellon University, Pittsburgh, Pennsylvania 15213, United States; § Department of Mechanical Engineering, Carnegie Mellon University, Pittsburgh, Pennsylvania 15213, United States

**Keywords:** sulfuric acid, sulfur trioxide, nocturnal chemistry, aerosols, steel emissions, coal burning, oxidation, particulate metals

## Abstract

Measurements of sulfuric acid (H_2_SO_4_) and sulfur trioxide (SO_3_) were conducted in Pittsburgh, Pennsylvania, during field campaigns in Fall 2023 and Fall 2024. These measurements identified nocturnal concentrations of H_2_SO_4_ comparable to those of daytime values. Nocturnal H_2_SO_4_ concentrations were observed to increase by 5 × 10^5^ to 5 × 10^7^ molecules cm^–3^ above background on 16 of the 31 measurement nights. The median peak concentration during events was 6.5 × 10^6^ molecules cm^–3^, with a maximum of 1.0 × 10^8^ molecules cm^–3^, exceeding previously reported nighttime concentrations. Increases in H_2_SO_4_ concentrations were positively correlated with the anomalously high SO_3_ concentrations and condensation sink rates, indicating that the formation of H_2_SO_4_ increased to overcome the loss rates to particles. Increases in particulate mass and the mass fraction of metals commonly emitted from coal combustion and steel production were also observed. The air masses were traced back to the southeast of Pittsburgh, a region home to a steel mill, coke plant, and a steel processing plant. The observations indicate a previously unrecognized nighttime formation pathway for H_2_SO_4_, potentially from heterogeneous catalysis with metal or black carbon, originating from steel and coke plant emissions. Further measurements are needed to identify key compounds and chemical processes driving these increases in nocturnal H_2_SO_4_ concentrations.

## Graphical abstract

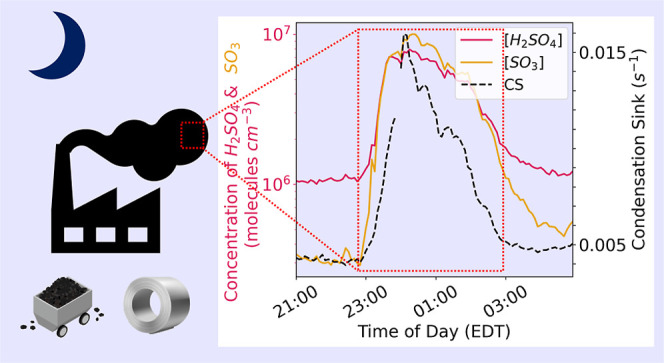

## 1 Introduction

Atmospheric sulfuric acid vapor plays an important role in the Earth’s radiative balance through new particle formation (NPF) and particle growth.^1–5^ NPF consists of the formation of stable particles via the reactions of gases and the subsequent growth of these particles to larger sizes. NPF processes produce roughly 50% of the global number of cloud condensation nuclei (CCN) and thus affect cloud formation and properties.^1–3,6^ Previous field observations have shown that atmospheric sulfuric acid vapor (H_2_SO_4_) is the key compound for NPF in most regions of the world.^7–9^ H_2_SO_4_ vapor also changes atmospheric particle composition and size through its condensation onto aerosol particles.^4,5^ However, uncertainty in modeling H_2_SO_4_ concentrations affects the predicted atmospheric aerosol concentration and composition.^1,2^

One contributing factor to the uncertainty in H_2_SO_4_ concentrations is the difficulty in modeling its formation pathways and rate of accumulation in the atmosphere.^10,11^ During the day, the formation pathway for H_2_SO_4_ is driven by photochemistry. Sulfur dioxide (SO_2_) is oxidized by hydroxyl radicals (OH), which are formed through the photodissociation of ozone (O_3_), nitrous acid (HONO), and formaldehyde (HCHO).^10,12–15^ As OH oxidizes SO_2_ to form sulfur trioxide (SO_3_), SO_3_ is rapidly converted to H_2_SO_4_ in less than a second via reactions with water^16^ and catalyzed by acids and ammonia.^17–19^ Daytime concentrations of H_2_SO_4_ span many orders of magnitude, with observations ranging from ∼1 × 10^5^ to 3 × 10^8^ molecules cm^–3^, while nocturnal concentrations are usually 10^6^ molecules cm^–3^ or below.^7,12,20^ However, recent field campaigns in Beijing, China, and Hyytiälä Forest, Finland, indicate that nonphotochemical sources can contribute to the formation of SO_3_ and H_2_SO_4_ at night and during the early morning.^12,20,21^ In Hyytiälä Forest during 2010, the maximum nighttime concentration of H_2_SO_4_ reached ∼1 × 10^7^ molecules cm^–3^.^20^ Despite the high nocturnal concentrations of H_2_SO_4_ observed in Hyytiälä, concentrations of H_2_SO_4_ generally decreased at night, with the highest nocturnal concentration observed at sunset. In Beijing, nocturnal increases in H_2_SO_4_ were attributed to its formation from stabilized Criegee intermediates (SCI) and a decrease in the condensation sink (CS), the loss rate of H_2_SO_4_ to preexisting particles.^12^ Criegee intermediates form when O_3_ reacts with alkenes.^12,20,22^ In the atmosphere, roughly half of the Criegee intermediates break down to form OH in less than a second^22^ with a small fraction forming dioxiranes and secondary ozonides.^23,24^ The other half is stabilized and forms SCI, which is capable of oxidizing SO_2_ directly into SO_3_ and ultimately H_2_SO_4_. Due to the short lifetime of H_2_SO_4_ in the atmosphere (∼1 min in a polluted urban environment), this compound was likely formed via nocturnal oxidation of SO_2_ and not from long-range transport.^12,21^

Catalytic oxidation of SO_2_ at the surface of traffic-related particles containing black carbon (BC) was also suggested to occur in Beijing during the early mornings when OH concentrations were low.^21^ In the winter, increasing SO_3_ concentration was observed in the early morning, suggesting that the formation pathway was not photochemically driven. These field measurements are supported by laboratory and computational studies that showed carbon-containing aerosols oxidizing SO_2_.^25^ The measurements from Beijing revealed that the formation of SO_3_ and H_2_SO_4_ correlated with the increased concentration of sub-2.5 nm particles in the early morning. As a result, aerosols containing BC induced the formation of H_2_SO_4_ and could ultimately influence the daytime particle concentration.^21^

This study presents nighttime observations of H_2_SO_4_ from Pittsburgh, PA located in Allegheny County. The nocturnal trends of H_2_SO_4_ exhibit distinctly different patterns from those observed in previous studies.^12,20,21^ Pittsburgh experiences a wide mixture of emissions typical of an urban environment with additional major contributions from the Mon Valley Works (MVW) complex. The MVW complex consists of a steel mill (Edgar Thompson Plant), a coke plant (Clairton Plant), and a steel processing plant (Irvin Plant), which are located 10–15 km to the southeast of Pittsburgh. The MVW complex has previously been reported to be a large source of SO_2_ and hydrogen sulfide (H_2_S).^26–28^ Specifically, the 2022 Allegheny County emission database showed that the Clairton Plant had the highest reported annual emissions of SO_2_, H_2_S, and particulate matter smaller than 2.5 μm in diameter (PM_2.5_) in Allegheny County.^27,28^ The Edgar Thompson Plant and Irvin Plant had the second and fourth highest sulfur oxides and PM_2.5_ emissions, respectively.^27^ In addition to sulfur compounds and PM_2.5_, extended measurements near the MVW complex and previous studies have shown coal burning can emit high concentrations of BC and trace metals, such as selenium, zinc, manganese, and iron.^29–33^

To better understand the sources of nocturnal H_2_SO_4_ in Pittsburgh, we measured atmospheric gas concentrations, particle size distributions, and particle composition. During the campaign, we consistently observed events where the concentrations of H_2_SO_4_ and SO_3_ increased during the night, with the median peak concentrations of 6.5 × 10^6^ and 9.0 × 10^6^ molecules cm^–3^, respectively, when resampled with a 5 min average. The maximum observed nighttime concentrations of H_2_SO_4_ and SO_3_ were 1.0 × 10^8^ and 1.6 × 10^8^ molecules cm^–3^, respectively, which are larger than any nighttime observations in Beijing or Hyytiälä.^12,20,21^ The nighttime measurements of H_2_SO_4_ during events were the same order of magnitude as daytime concentrations, which are known to impact the climate.^1,5,7^ Using measurements of trace atmospheric gases, particle composition, and meteorological conditions, we investigated the potential sources of air containing the increased H_2_SO_4_ concentrations and drivers of these events.

## 2 Materials and Methods

### 2.1 Carnegie Mellon University Sampling Site

The main measurement site was located on the Carnegie Mellon University (CMU) campus in Doherty Hall (see Figure S1 in the Supporting Information, SI). Two measurement campaigns were conducted: September 21st, 2023 to October 14th, 2023 and October 4th, 2024 to October 16th, 2024. The nighttime temperature ranged from 8 to 33 °C (mean of 17 ± 4.7 °C) with a relative humidity from 27 to 92% (mean of 64% ± 15%). During the 2023 campaign, instruments sampled from the third floor of the south side of the building, which overlooks a grassy quadrangle and a large park. In contrast, the instruments sampled from the first floor of Doherty Hall from a north-facing window during the 2024 campaign, see Figure S2. The north side of the building is more sheltered, with sections of the building to the east and west that extend past the window. The north side overlooks the building’s exhaust vents. Since the building is built on a hill, both the third-floor and the first-floor sample locations are ∼15 m above ground level.

H_2_SO_4_ and SO_3_ were measured by an atmospheric pressure interface long time-of-flight chemical ionization mass spectrometer (CIMS) with a custom-built transverse inlet using nitrate reagent ions (NO_3_^–^, HNO_3_·NO_3_^–^, and H_2_O·NO_3_^–^).^34–38^ Dominant signals for H_2_SO_4_ were HSO_4_^–^ and HNO_3_·HSO_4_^–^. For SO_3_, the observed signals included SO_4_^–^ and SO_3_·NO_3_^–^. Only the SO_3_·NO_3_^–^ signal was included in the conversion to concentration as SO_4_^–^ can also be formed from SO_2_ within the chemical ionization inlet.^21^ Signals were converted to concentrations following ionization kinetics and are detailed in the Supporting Information.^35,39^ The CIMS sampled at 15 LPM through an ∼100 cm long, 5 cm ID glass sample tube extending ∼50 cm outside the window. The chemical ionization reaction times were 0.018 and 0.016 s for the 2023 and 2024 campaigns, respectively. The selected chemical ionization reaction times allowed for the ionization of species such as H_2_SO_4_ and SO_3_ while limiting the products of chemical ionization reactions (and other ions produced by the radioactive source) from subsequent ionization of other atmospheric molecules. Ionization rate coefficients of 1.9 × 10^–9^ cm^3^ s^–1^ and 9.3 × 10^–10^ cm^3^ s^–1^ were used for H_2_SO_4_ and SO_3_ ionized by nitrate ions, respectively.^40,41^ In addition, CIMS signals were corrected for mass-dependent transmission efficiency measured on a similar instrument and diffusional wall loss for a similar glass sampling tube.^42–44^ The CIMS inlet and sampling flow were previously examined in the laboratory to ensure <30 s response time to a factor of 10–100 decrease in the H_2_SO_4_ concentration. Thus, the observations reported in this study reflect the changing sulfuric acid concentration within a 1 min time scale. See the Supporting Information for more discussion on the CIMS signal to concentration conversion.

During the campaigns, the 1.6–300 nm particle size distribution was measured by two stepping mobility particle sizing devices known as the particle sizing devices (PSD). The CS was calculated from the particle size distribution.^45–48^ The PSD sampled from a 10 cm ID community inlet, which operated at >250 LPM flow. The community inlet extended 1 m from the window and faced the same direction as the CIMS sample tube. More details on sampling for the PSD are provided in Cheng et al.^49^ Mass concentration of particulate matter smaller than 2.5 μm in diameter (PM_2.5_) was measured by a PurpleAir Flex sensor, located 250 m northeast of Doherty Hall, see Figure S2.

### 2.2 Supporting Observations at Lawrenceville, North Braddock, and Liberty

Additional gas and particle phase measurements were conducted by the Allegheny County Health Department (ACHD) and the Atmospheric Science and Chemistry mEasurement NeTwork (ASCENT) as part of permanent measurement sites in Pittsburgh.^50,51^ A map of all measurement sites is given in Figure S1. ACHD and ASCENT are located 3 km northwest of the CMU measurement site in Lawrenceville, PA. These measurements included SO_2_ (Teledyne API (TAPI) 100 EU), O_3_ (Thermo 49), NO_2_ (TAPI N500), NO_*y*_ (TAPI 200 EU), PM_2.5_ (Thermo RP Partisol-Plus 2025), BC (TAPI 633), and particulate metal concentration (Xact 625i). Ultraviolet radiation was measured with a Met One 094–1/6676. A Vaisala CL–51 ceilometer was used to determine the boundary layer height. The optimized Emeis method was used to calculate boundary layer height from the backscattering profiles.^52,53^ Additional gas monitors managed by ACHD were located in North Braddock, PA, and Liberty, PA, which are 10 and 15 km to the southeast of CMU, respectively. The North Braddock and Liberty monitors are near the MVW complex.

The ASCENT Site also included a 3839W89 scanning mobility particle sizer to measure the particle size distribution from 10 to 800 nm. This instrument was not operational during these campaigns, but its long-term observations showed particle concentrations above 300 nm of <10 particles cm^–3^. As a result, the CS determined from the PSD-measured 1.6–300 nm size distribution is representative of Pittsburgh air during the campaigns.

### 2.3 Plume Models and Citizen Science Measurements

Community-based observations were used to track the movement of polluted air masses and identify sulfur odors. Smell Pittsburgh (SmellPGH) is a mobile application where users report poor air quality, including the perceived severity, location, and an optional description of the smell.^54^ Reports were filtered to include only those within the Pittsburgh city limits, shown in Figure S1.

PM_2.5_ measured by ACHD and publicly available PurpleAir sensors were used to track the movement of plumes containing particles.^55^ The PM_2.5_ measurements were interpreted alongside Plume Pittsburgh, which uses the National Oceanic and Atmospheric Administration’s High-Resolution Rapid Refresh (HRRR) model to simulate plume transport from the MVW complex plants located southeast of the city.^56^

## 3 Results and Discussion

The CIMS measured frequent increases in the concentrations of nocturnal H_2_SO_4_ and SO_3_ during the 2023 and 2024 campaigns. Figure 1 shows examples of H_2_SO_4_ and SO_3_ concentration timelines for four nights with these nocturnal H_2_SO_4_ events. An event was defined by an increase in the H_2_SO_4_ concentration of more than 5 × 10^5^ molecules cm^–3^ or SO_3_ concentration of more than 3 × 10^5^ molecules cm^–3^ over any 30 min period between sunset and sunrise. This threshold was empirically chosen based on the distribution of increases in H_2_SO_4_ and SO_3_ across both campaigns. Examples of nonevent nights are shown in Figure S3. Over the course of 31 days of both campaigns, a total of 16 events were observed. Of the two campaigns, 2024 had a higher number of events, with an event occurring on all 12 nights. Sample event nights are shown in Figure 1 and exhibit an increase in H_2_SO_4_ and SO_3_ concentrations at all times of the night. For example, the event on October 5th, 2024 started shortly after sunset, whereas the events on October 5th, 2023 and October 6th, 2024 began around midnight and persisted past sunrise. The nighttime concentrations of H_2_SO_4_ and SO_3_ ranged from below detection limits of ∼2 × 10^5^ and ∼1 × 10^5^ molecules cm^–3^, respectively, to 1.0 × 10^8^ and 1.6 × 10^8^ molecules cm^–3^, respectively. Nocturnal H_2_SO_4_ concentrations during events were comparable to daytime values, which were generally on the order of 10^6^ to 10^7^ molecules cm^–3^. In addition to high peak concentrations, nocturnal events could persist for hours, with multiple events occurring on some nights, such as on October 11th–12th, 2023, which had events at 23:00 and 06:00 EDT.

**Figure 1:**
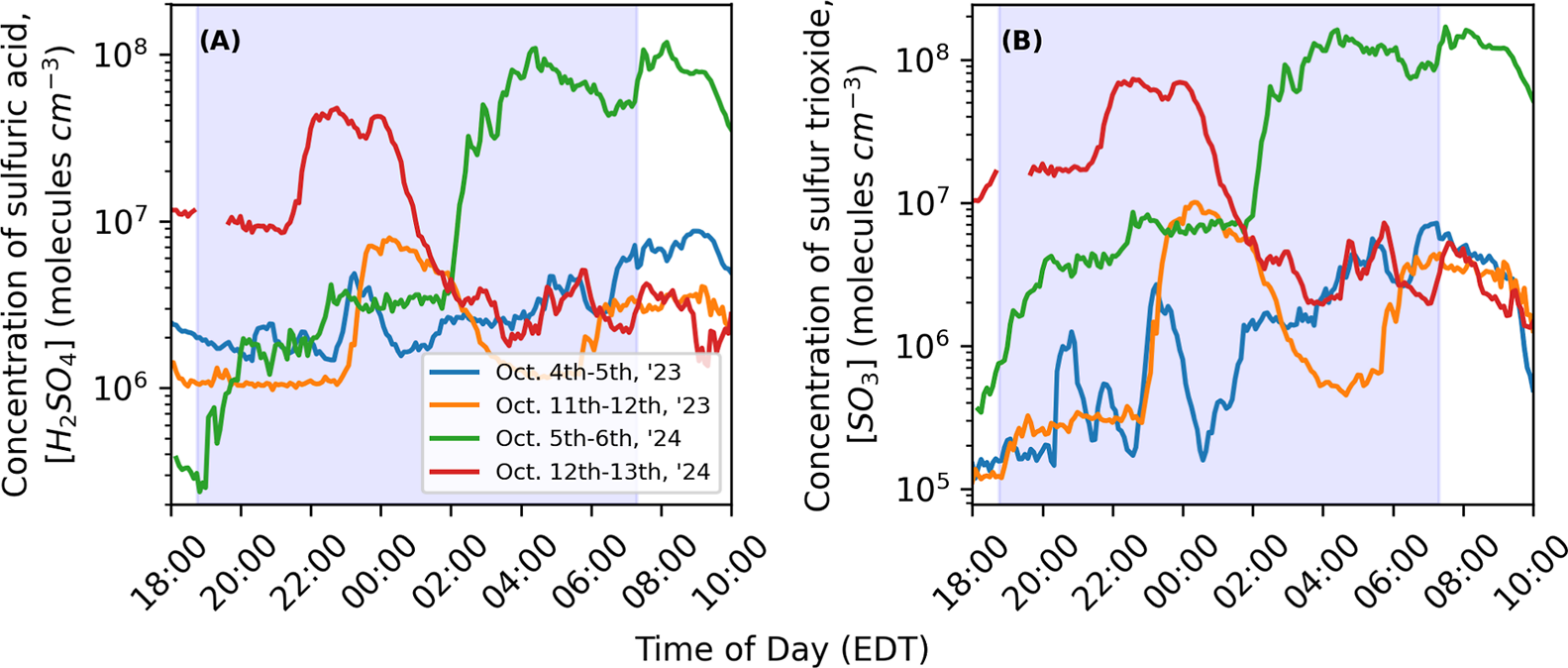
Example events where concentrations of (A) H_2_SO_4_ and (B) SO_3_ increased during the night in Pittsburgh, PA. Each color represents a different date, with shaded regions indicating the hours between sunset and sunrise.

Nocturnal background concentrations of H_2_SO_4_ were high at ∼2 × 10^6^ molecules cm^–3^ on most event and nonevent nights (Figure 1A), higher than those observed in Beijing (1 × 10^6^ molecules cm^–3^).^12,21^ Furthermore, background concentrations of SO_3_ were lower than H_2_SO_4_ in the Fall of 2023, often ∼2 × 10^5^ molecules cm^–3^ during both event and nonevent nights. During the Fall of 2024 event nights, background concentration of SO_3_ ranged from ∼2 × 10^5^ to 4 × 10^6^ molecules cm^–3^, which was higher than background H_2_SO_4_, which ranged from ∼1 × 10^5^ to 2 × 10^6^ molecules cm^–3^. Background concentrations of SO_3_ were higher in Pittsburgh than in Beijing, which had a median background concentration of 3 × 10^5^ molecules cm^–3^ during the winter.^21^

Although the background concentrations of H_2_SO_4_ and SO_3_ varied depending on the night, the changes in the concentration were strongly correlated. The Spearman correlation coefficient of SO_3_ with H_2_SO_4_ for each night across both campaigns is shown in Figure S4, where 14 out of the 16 event nights exhibited correlation coefficients greater than 0.9. The consistent correlation indicates that the formation of H_2_SO_4_ is driven by the hydrolysis of SO_3_, which aligns with previous observations of nighttime H_2_SO_4_.^12,20,21^ The observed high concentrations of SO_3_ on event nights also imply that SO_3_ and H_2_SO_4_ are being formed near the CMU measurement site, as SO_3_ is converted to H_2_SO_4_ in the atmosphere in less than 1 s and cannot be transported long distances.^16,21^

The ratio of SO_3_ to H_2_SO_4_ during these nighttime events ranged from 0.2 to ∼2 similar to those observed during polluted nights in Beijing.^12,21^ This ratio is dramatically higher than what was observed during the daytime, which ranged from ∼0.3 to below the detection limit (see Figure S5), and nights without events (∼0.2, see Figure S3). However, unlike Beijing with maximum SO_3_ concentrations of 1.7 × 10^6^ molecules cm^–3^, the Pittsburgh SO_3_ concentrations reached up to 10^8^ molecules cm^–3^. SO_3_ rapidly reacts with water which implies that the high SO_3_ concentration will result in an extremely fast production rate of H_2_SO_4_.^16^ For example, on October 12th, 2024 (Figure 1), the peak SO_3_ concentration was approximately 7 × 10^7^ molecules cm^–3^ with a temperature of 15.5 °C and a dew point of 5 °C. This translates to a H_2_SO_4_ production of ∼10^12^ molecules cm^–3^ s^–1^ (see Lovejoy et al.).^16^ This production rate far exceeds the observed H_2_SO_4_ concentration of 4 × 10^7^ molecules cm^–3^ even when taking into account a H_2_SO_4_ loss to pre-existing particles of ∼0.01 s^–1^. In contrast, the SO_3_ concentration observed during the day was much lower, typically below the detection limit of ∼1 × 10^5^ molecules cm^–3^. As a result, the anomalously high observed SO_3_ concentration must also reflect unknown chemistry either in the atmosphere or in the nitrate CIMS inlet, which would only enhance the signal at SO_3_·NO_3_^–^ measured by the CIMS at night.

Regardless of the accuracy of SO_3_ concentrations, the clear trends in increasing H_2_SO_4_ and SO_3_ signals during the event nights could be the result of several phenomena. One phenomenon is the contracting atmospheric boundary layer, which reduces the volume in the boundary layer and increases the concentration and coagulation of particles. The boundary layer typically collapses shortly after sunset. For example, on October 11th, 2023 as shown in Figure 2, the boundary layer height was ∼400 m before sunset and rapidly reduced to ∼80 m between 19:00 and 20:00 EDT. This effect can be seen in the particle size distribution shown in Figure 2A between 18:30 and 19:30 EDT on October 11th, 2023. The total concentration of particles rapidly increased with the mode diameter shifting from 15 nm at 18:30 to 26 nm at 19:30 EDT. However, the H_2_SO_4_ events depicted in Figure 2B occur more than two hours after the boundary layer collapsed. As a result, the changing boundary layer is not the cause of these H_2_SO_4_ and SO_3_ events.

**Figure 2:**
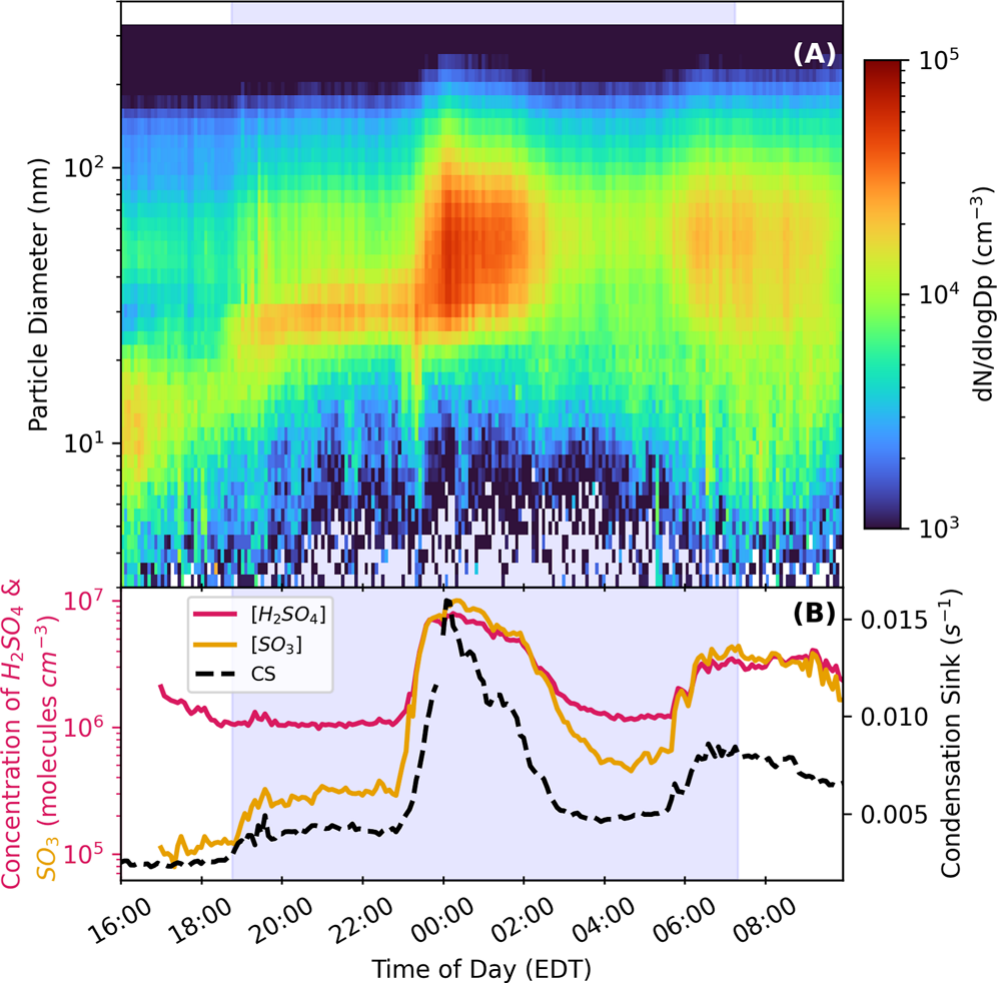
(A) Particle size distribution and (B) H_2_SO_4_ and SO_3_ concentration, and condensation sink (CS) for October 11th–12th, 2023.

The increase in H_2_SO_4_ at night could also be explained by a decrease in CS, the loss rate of H_2_SO_4_ to preexisting particles, combined with a constant formation rate of H_2_SO_4_ by SCI. In Beijing, the H_2_SO_4_ events had a negative correlation with CS. Figure 2 demonstrates that concentrations of 20–200 nm aerosol particles and their mode diameter (from 30 to 50 nm) increased with increasing H_2_SO_4_ as seen in Figure 2 at 23:00. The increase in size and concentration translates to a higher CS,^12,45,46^ which correlates well with H_2_SO_4_ and SO_3_ concentrations (see Figure S4) but anticorrelates during the day (Figure S5). The high CS at night likely explains why the increase in H_2_SO_4_ did not result in the formation of new particles (Figures 2 with more discussion in the Supporting Information). In contrast to the observations from Beijing, the positive correlations of H_2_SO_4_ with CS at night in Pittsburgh imply that the formation rate of H_2_SO_4_ must increase to compensate for the increased loss rate. Furthermore, H_2_SO_4_ and SO_3_ must be formed near the sampling site, as both compounds have short lifetimes (less than 100 and 1 s, respectively) due to the high CS loss rate.^21,46–48^

One way the formation rate increases is if the concentration of SCI increased during the events. From Guo et al., the formation rate of H_2_SO_4_ from SCI can be approximated as *k*_app_[SO_2_]­[O_3_]­[Alkenes], where *k*_app_ is an empirically fitted rate coefficient ranging from 1.5 × 10^–30^ to 2.7 × 10^–30^ cm^6^ s^–1^.^12^ The concentrations of alkenes were not measured during the Pittsburgh campaigns. To estimate the required alkene concentration to form the H_2_SO_4_ concentrations observed during the events, the steady-state H_2_SO_4_ chemical balance from Guo et al. can be solved for the alkene concentration (eq 1), where β is the hard-sphere collision rate between H_2_SO_4_ molecules with a value of 3.47 × 10^–10^ cm^3^ s^–1^.^12^

1 $$\left[Alkenes\right]=\frac{\left(CS\left[H_{2}{\text{SO}}_{4}\right]+{\beta \left[H_{2}{\text{SO}}_{4}\right]}^{2}\right)}{k_{app}\left[{\text{SO}}_{2}\right]\left[O_{3}\right]}$$

On October 11th and 12th, 2023 (Figure 2) from 22:00 to 4:00, the [SO_3_] and [O_3_] were measured at a maximum of 0.6 and 1 ppb, respectively, in Lawrenceville. As such, the calculated concentration of alkenes needed to explain the rise in the observed H_2_SO_4_ concentrations is 1 × 10^11^ molecules cm^–3^ at 22:00 to 7 × 10^13^ molecules cm^–3^ at midnight. This predicted concentration is likely much higher than that in Pittsburgh. For example, the Environmental Protection Agency Photochemical Assessment Monitoring Station in Pittsburgh measured concentrations of propylene during the summer of 2023, with a maximum observed concentration of 8 × 10^10^ molecules cm^–3^.^57^ Older studies from Pittsburgh have presented the maximum ethylene concentration of 3 × 10^10^ molecules cm^–3^,^58^ propylene of 7 × 10^9^ molecules cm^–3^,^59^ and butene of 5 × 10^9^ molecules cm^–3^.^59^ The highest alkene concentration observed in Beijing was ∼4 × 10^12^ molecules cm^–3^, and the concentration did not change by more than an order of magnitude on any night.^12^ Alkene concentrations would have to be orders of magnitude higher than previously observed to compensate for the increased CS during events, so this analysis suggests that SO_2_ oxidation by SCI is unlikely to explain the H_2_SO_4_ events.

The strong positive correlation of SO_3_ and H_2_SO_4_ concentrations with CS coupled with the sudden appearance of particles suggests that these compounds must be forming inside a particle-laden plume that travels to the measurement site at night. The wind velocity was used to determine where the plume was emitted. During the campaign, the wind speed was low (0.8–1.5 m s^–1^) on both nonevent and event nights, further supporting the argument that the H_2_SO_4_ was not transported long distances. In addition, the nocturnal boundary layer consistently contracted after sunset to ∼100 m, which reduced the volume of the boundary layer and made local emissions more pronounced. On event nights, the wind direction measured at the Lawrenceville and North Braddock ACHD sites blew from the southeast while the Liberty ACHD sites measured a southerly wind direction. This contrasts with nonevent nights where the wind at Lawrenceville, North Braddock, and Liberty most often came from the north, east, and east northeast, respectively. The low wind speeds at night may affect measurements of wind direction, which have increased uncertainty when the air is calm.

To further pinpoint the source of the plume, PurpleAir PM_2.5_ and ACHD PM_2.5_ measurements were used to track the plume’s movement through the region. PM_2.5_ was used as it correlates with CS, H_2_SO_4_, and SO_3_ (more details on this below). Figure 3 shows the hour when each PM_2.5_ sensor first observed the air mass containing elevated PM_2.5_ from October 11th to 12th, 2023. The timing corresponds to the one hour period with the largest increase in the PM_2.5_ concentration at each sensor, excluding any sensors with a nightly maximum concentration less than 3 μg m^–3^. Note, observations near Lawrenceville often increased earlier in the night than other PM_2.5_ sensors in Pittsburgh, indicating that Lawrenceville may sample a different air mass or measure PM_2.5_ from a hyperlocal source. In Figure 3, a large increase in PM_2.5_ is observed near the MVW complex plants between 21:00 and 22:00 EDT, suggesting that this area is the source of the plume. The air mass then moved toward Pittsburgh, with sensors near CMU observing the largest increase in PM_2.5_ between 23:00 and 01:00 EDT. This time aligns with the first H_2_SO_4_ event of the night (Figure 2), further supporting the conclusion that H_2_SO_4_ is from the plume. In addition, Gaussian plume models from Plume Pittsburgh show potential emissions from the MVW complex sites blowing toward Pittsburgh and reaching CMU between 23:00 and 23:59 EDT see Figure S7. The plume model also shows that the Lawrenceville site experienced the plume later than CMU did. The combined information from PM_2.5_ measurements and plume modeling reveals that the air mass containing PM_2.5_, H_2_SO_4_, and SO_3_ comes from the region southeast of the measurement site where the MVW complex is located.

**Figure 3:**
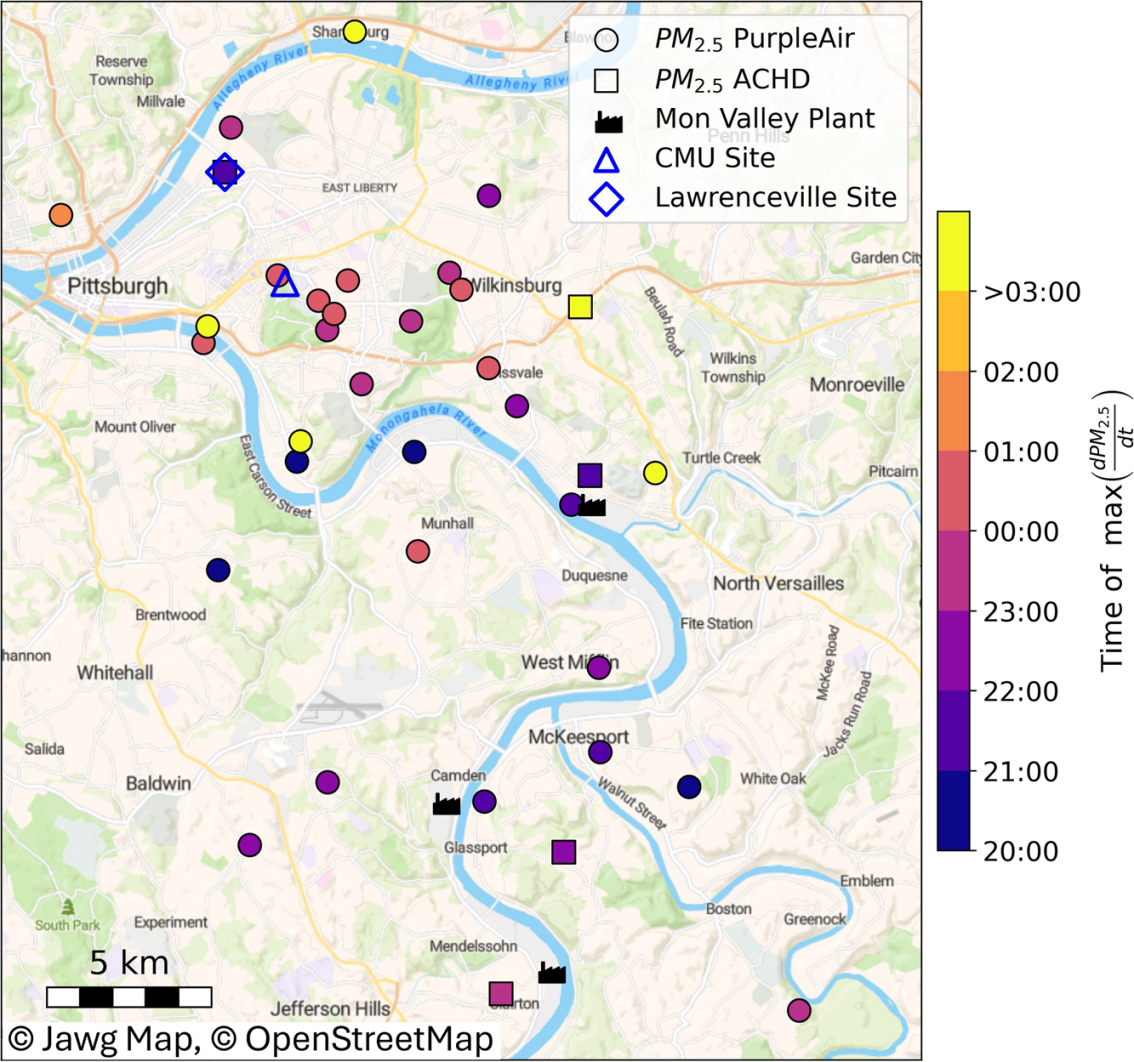
Map of Pittsburgh and its surrounding regions. Points are colored based upon the time of max increases in PM_2.5_ between the hours of 20:00 and 07:00 on October 11th–12th, 2023. Circles are PM_2.5_ measurements from PurpleAir sensors, and squares are from ACHD sensors. Map is reproduced with permission from Jawg Maps.

The Lawrenceville site contained additional instruments for gas and particulate composition that complement the measurements conducted at the CMU. In Figure 4, gas and particle phase measurements from the nights of October 11th–12th, 2023 (A–C) and October 12th–13th, 2024 (D–F) taken from both locations. To relate these measurements with those at CMU (Figure 4A and D), the plume was first confirmed to be present at both locations at the same time. Figure 4B and E displays the concentrations of PM_2.5_ measured at CMU and Lawrenceville. As seen with the CS, the concentrations of PM_2.5_ near CMU positively correlated with H_2_SO_4_ for both nights. The concentration of PM_2.5_ in Lawrenceville increased early in the night for both nights, likely due to hyperlocal sources, making it difficult to identify when the particle-filled plume reached Lawrenceville. However, the mass fraction of BC, a tracer for combustion emissions,^25,29^ in Lawrenceville increased at 23:00 EDT on October 12th, 2024 (Figure 4E). This trend is also seen in the early morning (06:00) on October 12th, 2023 (Figure 4B), when the second H_2_SO_4_ event of the night is observed. It is likely that the CMU and Lawrenceville sites are both measuring the same air mass in the early morning of October 12th, 2023; however, it is obscured in Figure 3 by emissions from earlier in the night.

**Figure 4:**
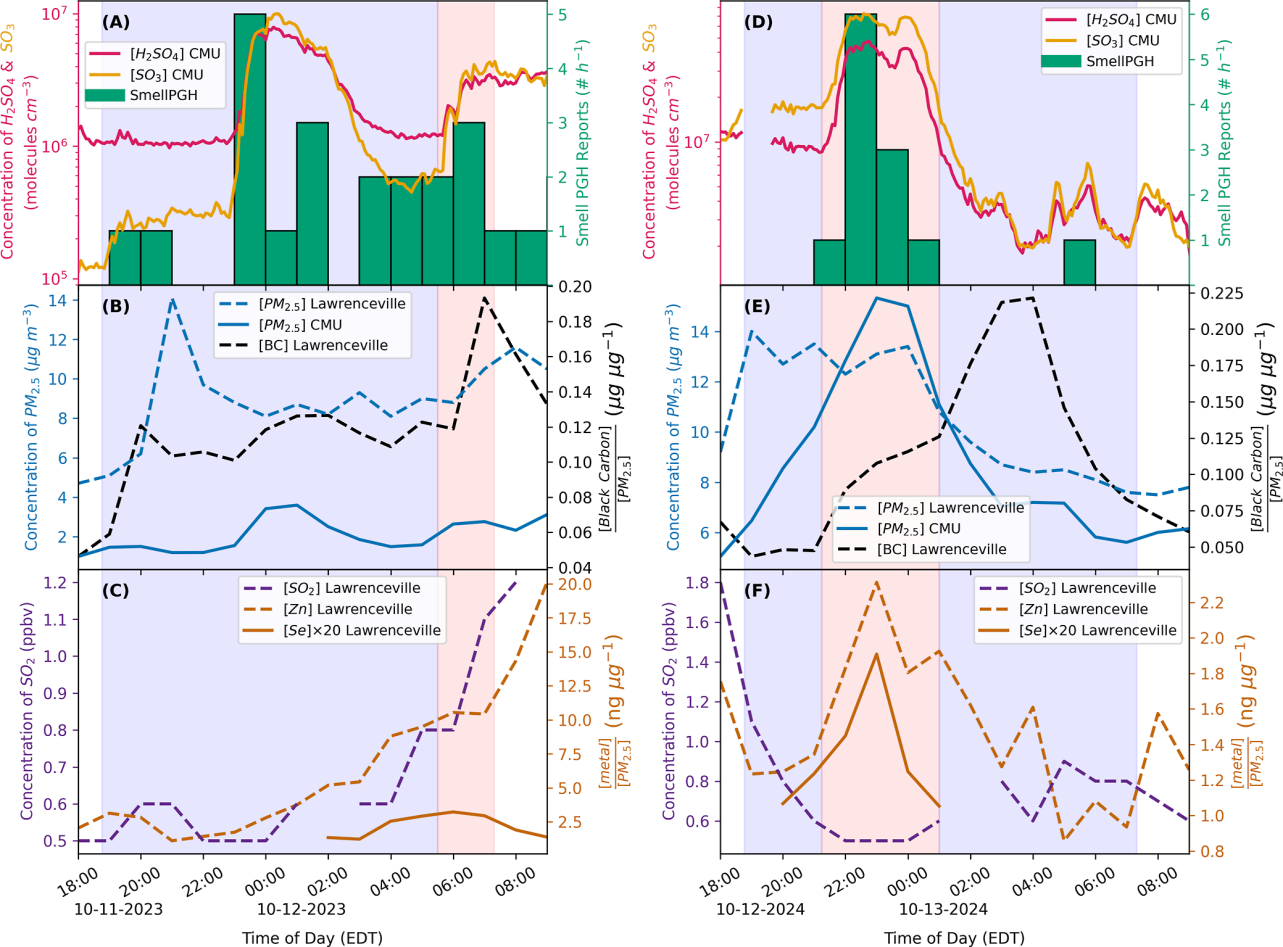
Measurements during two H_2_SO_4_ events on October 11th–12th, 2023 (A–C) and October 12th–13th, 2024 (D–F). H_2_SO_4_ and SO_3_ were measured at CMU, and SmellPGH reports were from within Pittsburgh’s city limits (A and D). Black carbon (BC) was observed at Lawrenceville, and PM_2.5_ was measured at CMU and Lawrenceville (B and E). SO_2_ concentration and mass fraction of metal to PM_2.5_ were taken at Lawrenceville (C and F). The purple region indicates nighttime hours with the overlapping pink shaded region signifying times the plume was likely sampled at the CMU and Lawrenceville sites.

The strong correlation between H_2_SO_4_ and SO_3_ with BC does not hold when the plume reaches the sites at different times. For example, between 23:00 and 00:00 EDT on October 11th to 12th, 2023 (Figure 4B), the PM_2.5_ concentration and BC mass fraction do not clearly increase with the H_2_SO_4_ and SO_3_ observed at CMU. Figure 3 and the plume modeling (Figure S7) demonstrate that the first plume reaches CMU at 00:00 EDT on October 12th, 2023; however, the plume model then shows that the plume trajectory changed direction before reaching Lawrenceville. A lower concentration of emissions may still reach Lawrenceville around 00:00 EDT on October 12th, which is consistent with the small increases in BC, PM_2.5_, and metals at Lawrenceville. In addition, BC mass fraction and H_2_SO_4_ are anticorrelated on October 13th, 2024 between 01:00 and 02:00 EDT, during which the PM_2.5_ concentration at both sites also decreases. The increase in BC mass fraction indicates a change in the particle emission source from the plume originating near the Mon Valley. This also resulted in a decrease in the formation of SO_3_ and H_2_SO_4_ at the CMU.

To better constrain the source of the plume, particulate metals measured at Lawrenceville were examined during periods when the plume reached CMU and Lawrenceville. As shown in Figures 4C and F and S9, mass fractions of Zn, Se, Fe, and Mn were observed to increase during the events on October 11th–12th, 2023, and October 12th–13th, 2024. This further indicates emissions from coal burning, as all three metals can be emitted by coal combustion.^30–32,60^ Similar to BC, the correlation of metal mass fraction with the H_2_SO_4_ concentration (Figure S10) varied widely depending on the night, likely a result of Xact and CIMS measuring different air masses.

The combined observations across these two sites demonstrate two important points: (1) Lawrenceville measured the same air plume as CMU at 06:00 on October 12th, 2023, and 22:00 on October 12th, 2024, as the sites observed a simultaneous increase in BC, metals, PM_2.5_, H_2_SO_4_, and SO_3_ and (2) the plume originated from coal combustion sources. This further supports that the plume was emitted from the region with the MVW complex, where the coke plant, steel mill, and steel processing plant are known producers of high concentrations of PM_2.5_, BC, and trace metals.^32,33,60,61^

The production of SO_3_ and H_2_SO_4_ requires precursor gases of either SO_2_ and/or H_2_S. Figure 4A and D demonstrates that the timelines of SmellPGH reports across the city correlate with increases in concentrations of H_2_SO_4_ and SO_3_. All SmellPGH reports originate from users in Pittsburgh who self-report unpleasant smells or bad air quality. Of the SmellPGH reports, 24% contained optional keywords related to the smell of sulfur (e.g., rotten eggs, brimstone, and sewage). The sulfur smells reported are more likely H_2_S than SO_2,_ as the smell of H_2_S is recognizable at five ppbv, while SO_2_ is not detectable by smell until one ppmv.^62,63^ Methanethiol is another potential sulfur-smelling compound; however, this compound has not previously been reported as an emission from steel production or coke works. In addition, the ACHD North Braddock and Liberty sites, see Figure S1 for locations, measured nocturnal H_2_S concentrations up to 20 ppbv (10/11/2024) and 52 ppbv (10/1/2023), respectively, when the plume traveled toward these ACHD sites instead of northwest to CMU. These observations indicate that the plumes contain high concentrations of H_2_S. As such, the H_2_SO_4_ and SO_3_ events are likely linked to an increase in H_2_S concentrations.

Concentrations of SO_2_, the key precursor for H_2_SO_4_ and SO_3_, measured in Lawrenceville are shown in Figure 4C and F. The concentration of SO_2_ remained low during both campaigns. Most nightly maxima fall below one ppbv, lower than the median SO_2_ concentration of two ppbv observed during nocturnal events in Beijing.^12,21^ Beijing events saw a positive correlation between SO_2_, H_2_SO_4_, and SO_3_, indicating SO_2_ oxidation is the source of nighttime SO_3_ and H_2_SO_4_ formation. During Pittsburgh campaigns, the median correlation between nightly SO_2_ and H_2_SO_4_ concentrations, displayed in Figure S4, was 0.10 for the event nights. The weak correlation may be due to differences in the air masses at the different sampling locations, an unidentified limiting reagent in the oxidation processes, or a combination of both. During the event on October 12th, 2024, when both CMU and Lawrenceville are expected to sample the same air mass, the correlation between SO_2_ and H_2_SO_4_ was negative, suggesting that low concentrations of SO_2_ (∼0.5 ppbv) do not limit the nocturnal formation of H_2_SO_4_. Measurements of SO_2_ concentration collocated with H_2_SO_4_ are needed to rule out the impacts of location and dilution on the measurements, as the insensitivity to SO_2_ concentration contrasts with previous observations of nocturnal H_2_SO_4_ formation.^12,21^

In a study by Yuo et al., the formation of nocturnal SO_3_ was explained by the heterogeneous catalysis of SO_2_ on the surface of BC aerosol particles. They observed a positive correlation between concentrations of SO_3_ and the product of SO_2_ and BC concentrations ([SO_2_]×[BC]). To examine if this is a potential nighttime H_2_SO_4_ and SO_3_ formation pathway for Pittsburgh, [SO_2_] × [BC] were correlated to the concentrations of SO_3_ and H_2_SO_4_ (Figure S4). For event nights, there was no overall correlation as the median correlation was 0.03. During some time periods, such as 06:00 on October 12th, 2023, SO_2_, BC, H_2_SO_4_, and SO_3_ all increased at the same time. However, the overall correlation of [SO_2_] × [BC] for October 11th to 12th, 2023 was 0.03 as it did not correlate with the first H_2_SO_4_ event of the night. Measurement of BC and SO_2_ at CMU would likely improve the correlation but may not explain all events due to low SO_2_ concentrations when both sites measured the same air mass, as discussed previously. As such, catalytic formation of H_2_SO_4_ from BC and SO_2_ does not seem to be the dominant pathway for nocturnal H_2_SO_4_ formation in the steel and coal emission plume.

In addition to BC, metal oxides have been shown to act as catalysts for the heterogeneous oxidation of SO_2_ and H_2_S and are used in the industrial production of H_2_SO_4_.^64–68^ A wide range of metal oxides can act as catalysts, including oxides of Fe, Mn, and Zn.^64,65,67–69^ Although the Xact is not able to measure the oxidation state, these metals were observed during events (Figures 4 and S9) and have previously been observed to exist in high concentrations as oxides in submicron coal combustion particles.^31,67,70^ In addition, mixtures of these metal oxides, which have been identified in emissions from steel making, may be more effective at oxidizing SO_2_ than oxides containing one type of metal.^70^ Oxidation of H_2_S may help explain the events, as concentrations of SO_2_ measured in Lawrenceville were low, with most nights below one ppbv. The coke plant and steel mill are known to emit H_2_S, and Pittsburgh residents frequently report smells associated with H_2_S during the night.

Although not previously observed in field measurements, oxidation reactions catalyzed by metal oxides can occur at ambient conditions, with laboratory measurements of oxidation of H_2_S and SO_2_ observed at room temperature on a ternary metal oxide containing nanoparticles of Fe, Mn, and Zn.^66^ However, the oxidation of sulfur compounds on the ternary metal oxides did not observe desorption of the product from the oxide surface to the gas phase, which has a high activation energy at low temperatures.^65^ For aerosols containing metal oxides, the size and curvature of the particles may decrease the energy required for desorption, resulting in more desorption from the aerosol surface at a lower temperature. These effects are dependent on the adsorbed molecule, catalyst composition, particle size, and temperature.^71–73^ Other properties of the aerosol may enhance the oxidation rate, such as the presence of binary metal oxides, higher concentrations of metals at the surface of the coal combustion particle, and a higher concentration of reaction sites compared to bulk.^64,74,75^ To determine the oxidation state of the metals, samples of PM_2.5_ should be examined by X-ray photoelectron spectroscopy.^76^ Computation chemistry studies, similar to those done for SO_2_ and BC aerosols,^25^ may help identify if the adsorption, oxidation, and desorption of H_2_S and SO_2_ on metal oxide aerosols is a feasible nocturnal formation pathway for SO_3_ and H_2_SO_4_ at atmospheric conditions.

Another family of compounds that may enhance the oxidation of sulfur molecules are reactive nitrogen compounds (e.g., NO_*x*_, NO_*z*_, and HONO), which can be emitted by coal combustion and steelmaking.^77–79^ NO_*x*_ concentrations measured in Lawrenceville showed a strong correlation during event nights when the plume reached CMU and Lawrenceville, such as on October 12th–13th, 2024 with an observed correlation of 0.79. In addition, nitrate (NO_3_^–^, formed from hydrolysis of N_2_O_5_ at night)^80^ has previously been shown to improve uptake and accelerate oxidation of SO_2_ on Fe_2_O_3_ at ambient temperatures and in the absence of solar radiation.^81^ The CIMS also observed 93.00, 108.99, and 140.98 amu to correlate with H_2_SO_4_ and SO_3_ (correlation values of 0.50, 0.42, and 0.49 during event nights, Figure S11). Though definitive identification of these peaks is challenging, the masses and isotope ratios correspond to nitrogen oxides, specifically HNO·NO_3_^–^, HONO·NO_3_^–^, and HNO_4_·NO_3_^–^, see the Supporting Information for more discussion. The increase of HONO-related masses could also be explained by nitrate enhancement of SO_2_ on Fe_2_O_3_ as the process was previously found to form HONO.^81^ The strong correlation of the reactive nitrogen compounds could suggest that reactive nitrogen compounds emitted from coal combustion and steelmaking may enhance the uptake of SO_2_ onto metal oxides and accelerate the oxidation of SO_2_ into SO_3_ and H_2_SO_4_.

Overall, measurements and models conducted during the campaigns show air masses on H_2_SO_4_ event nights originating from the southeast of Pittsburgh near the MVW complex. The air plumes are likely emissions from the steel industry, as these plants are the largest emitters in that area. The H_2_SO_4_ events occurred on a local scale with the different parts of Pittsburgh observing the plume at different times. The measured air mass contained elevated BC and trace metal concentrations, further suggesting that they contain emissions from combustion. Previous studies suggest that metal oxides and BC could heterogeneously oxidize H_2_S and SO_2_ into SO_3_ and H_2_SO_4_. Observations of the polluted air masses in Pittsburgh demonstrate that metal oxides, such as those from Fe, Mn, Zn, and BC are present in the aerosol particles and correlate well with H_2_SO_4_ events. In addition, the very high correlation of H_2_SO_4_ and SO_3_ with reactive nitrogen compounds (e.g., HONO) suggests that these compounds could potentially accelerate the uptake and oxidation of SO_2_ with Fe_2_O_3_ on the surface of aerosol particles. These observations of nocturnal SO_3_ and H_2_SO_4_ in Pittsburgh demonstrate previously unobserved chemistry occurring in the emissions from coal burning or steel production. More laboratory and collocated field measurements are needed to quantify the various potential reaction pathways and identify key compounds in the nighttime oxidation of sulfur species to SO_3_ and H_2_SO_4_ within the emissions plume.

## Supplementary Material



## Data Availability

Measurements from the CMU site are available at 10.1184/R1/30577211. Data from the ACHD is available on the Western Pennsylvania Regional Data Center (https://data.wprdc.org/dataset/allegheny-county-air-quality). PurpleAir measurements can be downloaded using the PurpleAir api (https://api.purpleair.com/). Measurements from ASCENT will be available in the future on their own database, see https://ascent.research.gatech.edu/database for more information. Plume Pittsburgh and SmellPGH are available through the CMU Create Lab (https://www.cmucreatelab.org/projects).

## Notes

Further descriptions of measurement sites, CIMS, nonevent night timelines, nightly correlations of H_2_SO_4_, H_2_SO_4_ 24 h timeline, Plume Pittsburgh Gaussian Plume Models of MVW emissions, Xact PM_2.5_ metal composition, and CIMS *m*/*z* ratios correlated with H_2_SO_4_ (PDF)
