# Supplementary material for: Observations of Nocturnal Sulfuric Acid Formation in Pittsburgh, PA
Source: Environ Sci Technol. 2026 Apr 10;60(16):12351–61. doi: 10.1021/acs.est.5c14137 (PMC13130960; doi:10.1021/acs.est.5c14137)
Supplement: Supplementary file 1 [file es5c14137_si_001.pdf]

Supplemental Information: Observations of Nocturnal Sulfuric Acid Formation in Pittsburgh, PA

Dominic A. Casalnuovo[1,2], Darren Cheng[2,3], Christine Troller[1,2], Ziheng Zeng[1,2], Albert A. Presto[2,3], *Coty N. Jen[1,2]

[1]Department of Chemical Engineering, Carnegie Mellon University, Pittsburgh, PA, 15213, USA
[2]Center for Atmospheric Particle Studies, Carnegie Mellon University, Pittsburgh, PA, 15213, USA
[3]Department of Mechanical Engineering, Carnegie Mellon University, Pittsburgh, PA, 15213, USA

*(cotyj@andrew.cmu.com)

1. Measurement sites

Figure S1 is a map of Pittsburgh and the surrounding suburbs with labeled measurement sites. The map also includes the location of the three plants that make up the Mon Valley Works (MVW). The distance between Lawrenceville and Carnegie Mellon University (CMU) measurement sites was 3 km. An overhead view of Doherty Hall and the surrounding buildings, where the CMU measurements were taken, is shown in Figure S2. Mass concentration of particulate matter smaller than 2.5 µm in diameter ($PM_{2.5}$) was measured from the second floor of the Robert Mehrabian Collaborative Innovation Center using a PurpleAir Flex sensor. The North Braddock and Liberty sites were 10 and 15 km from CMU, respectively. Pittsburgh and the surrounding area contain complex geography, including valleys and many hills, which are shown in Figure S1 using shading.

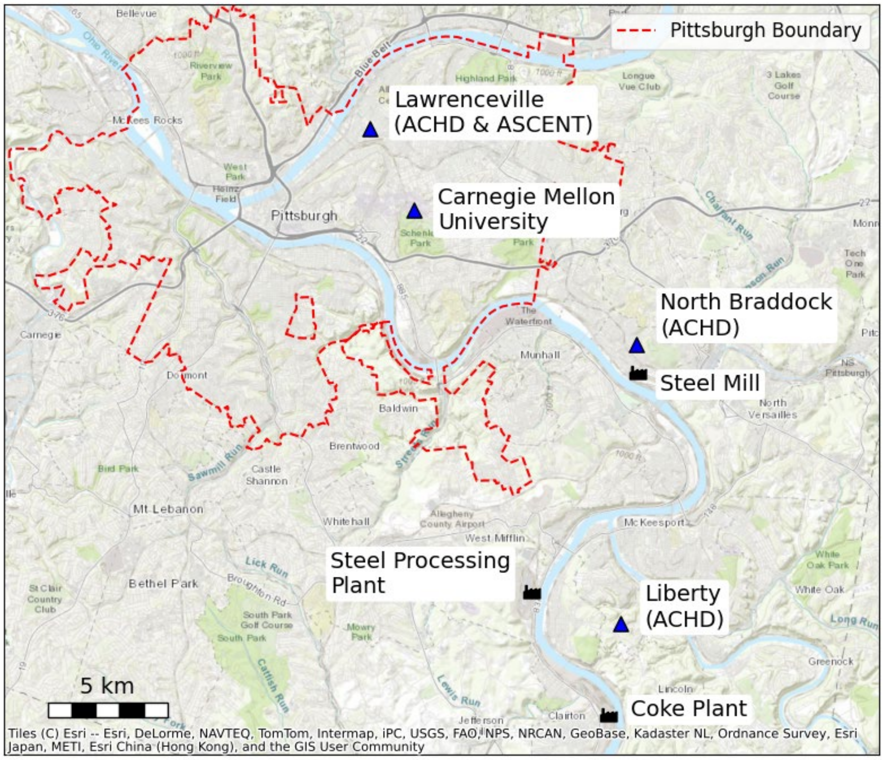

Figure S1. The locations of the measurement sites (blue triangles) and the three plants of the Mon Valley Works (factory symbol) are overlaid over a topographical map of the Pittsburgh area. Pittsburgh city limits are marked by a red dashed line.

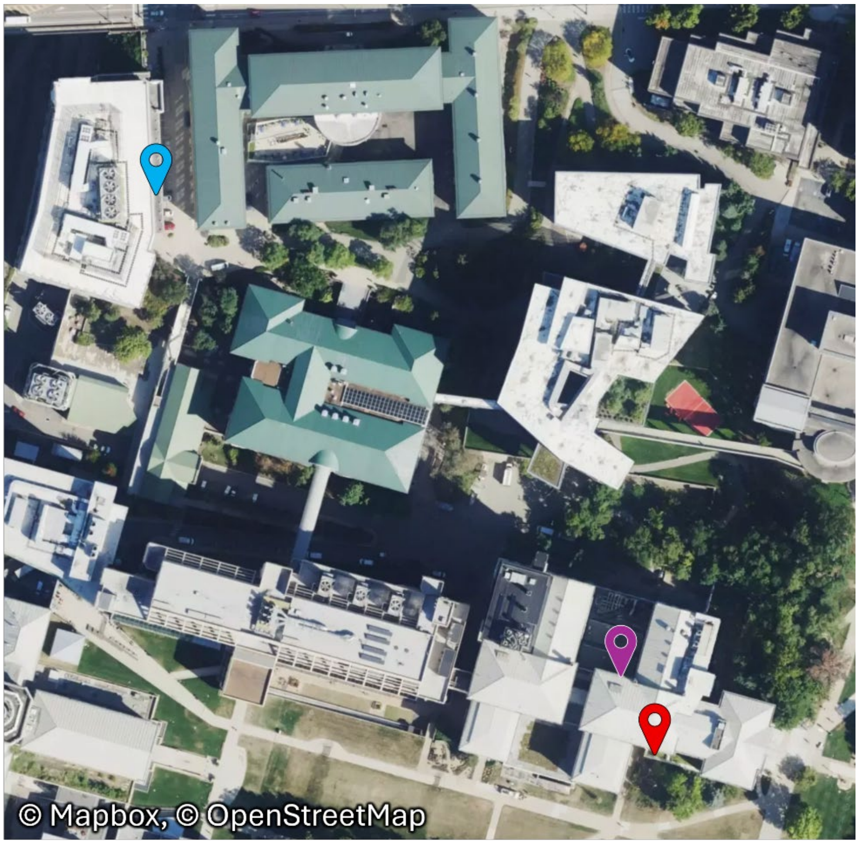

Figure S2. Overhead view of Doherty Hall (bottom right) and the surrounding buildings, where CMU measurements were taken. Measurements from the Fall 2023 campaign (red marker) were south facing, while the Fall 2024 campaign (purple marker) were north facing.[1] $PM_{2.5}$ concentrations for both campaigns were measured by PurpleAir sensor at the blue marker, 250 m northwest of Doherty Hall. Aerial is reproduced with permission from MapBox and OpenStreetMap.

2. Chemical Ionization Mass Spectrometer

The chemical ionization mass spectrometer (CIMS) charges neutral atmospheric molecules, such as H_2SO_4 and SO_3, using nitrate ions (NO_3^-, $HNO_3{\cdot}NO_3^-$). Dominant signals for H_2SO_4 were HSO_4^- and $HNO_3{\cdot}HSO_4^-$. For SO_3, observed signals included SO_4^- and $SO_3{\cdot}NO_3^-$. Only $SO_3{\cdot}NO_3^-$ was included in the conversion to concentration as SO_4^- can also be formed from SO_2 within the chemical ionization inlet.[2] Thus, the true concentration of SO_3 could be higher than reported, as signals of SO_4^- were similar in value to $SO_3{\cdot}NO_3^-$.

The conversion of the signal for species, i, to concentration is shown in Equation S1. z_i is the mass-dependent transmission efficiency, $k_{I,i}$ is the ionization rate coefficient, and t_{CI} is the chemical ionization time between the reagent and neutral atmospheric molecules.[3–5] For H_2SO_4 and SO_3, $k_{I,i}$ is 1.9×10^{-9} cm^3 s^{-1} and 9.3×10^{-10} cm^3 s^{-1}, respectively.[6,7]

$$\frac{Signal_i}{Signal_{reagent}} = z_i k_{1,i}[i]t_{CI} \qquad \text{Equation S1}$$

The t_{ci} is for the 2023 and 2024 campaigns were 0.018 and 0.016, respectively. This time was determined by assuming a parallel plate electric field in the transverse chemical ionization inlet. The distance between the source plate and front plate is 5 cm, the ion mobility for nitrate monomer reagent ion is 2.47 cm^2 s^{-1} V^{-1}, and the electric field strengths were set to 110 and 130 V cm^{-1} for the 2023 and 2024 campaigns, respectively.[8] The detection limit of H_2SO_4 was limited by interference from another nearby peak. At less than 1 ions s^{-1} of H_2SO_4, the analysis software was not always able to differentiate the H_2SO_4 peak from the tail of the interfering peak. This is not expected to impact measurements of H_2SO_4 as 1 ion s^{-1} is 2.4×10^5 molecules cm^{-3}, and concentrations at night were generally greater than 1×10^6 molecules cm^{-3}.

For SO_3, which did not have interfering peaks, signals as low as 0.3 ions s^{-1} were observed during the campaign, which corresponds to a concentration of ~1×10^5 molecules cm^{-3}.

3. Non-event night timelines of H_2SO_4 and SO_3

Non-event nights, which were defined as a night without an increase in concentration of more than 5×10^5 molecules cm^{-3} of H_2SO_4 or 3×10^5 molecules cm^{-3} of SO_3 over any 30-minute period between sunset and sunrise, were observed 15 times during the Fall 2023 campaign and 0 times during the Fall 2024 campaign. Figure S3(A) shows a non-event night where the concentration of H_2SO_4 and SO_3 saw no sharp increases, and the concentration of SO_3 remained close to the detection limit of the CIMS. The night of September 29th-30th, 2023 (Figure S3(B)) shows increases in H_2SO_4 and SO_3 concentrations; however, the changes were below the event classification threshold, and it was not classified as an event night.

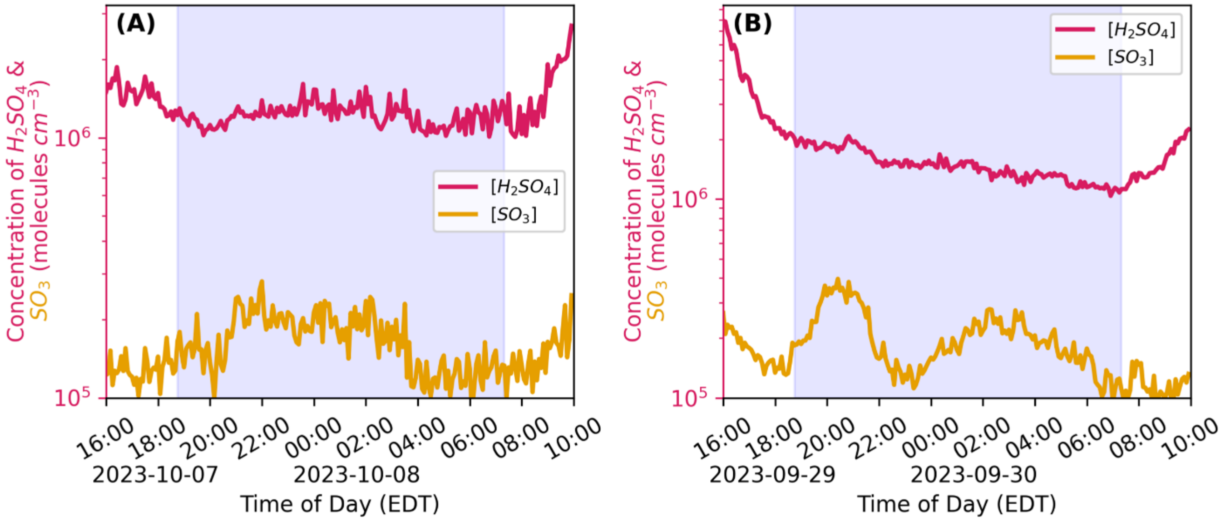

Figure S3. Measurements of concentration of H_2SO_4 and SO_3 at CMU during non-event nights on October 7th–8th, 2023 (A) and September 29th–30th, 2023 (B).

4. Observed nightly correlations with H_2SO_4

Figure S4 shows the Spearman correlation between H_2SO_4 concentrations and various measurements from CMU and ACHD Lawrenceville sites for each night during campaigns. Concentrations with no correlation, shown as white, result when all measured values are zero or when there were no measurements available. Measurements were resampled using one-hour averaging before correlation was calculated using the pandas correlation function in Python. Spearman correlation was selected as the relationships are often nonlinear. The largest correlations were with SO_3 concentration and condensation sink (CS), with median values of 0.95 and 0.79, respectively, on nights when H_2SO_4 concentration was greater than 2×10^6 molecules cm^{-3}. On these nights, $PM_{2.5}$ concentration near CMU had a median correlation value of 0.63, while Lawrenceville $PM_{2.5}$ had a score of -0.01. As discussed in the main paper, both differences in air masses and hyperlocal sources near Lawrenceville make it difficult to compare measurements from the two sites.

On non-event nights, the correlation between SO_3 and H_2SO_4 had high variance, with coefficients ranging from -0.66 to 0.93 (Figure S4). On the 4 non-event nights with correlation greater than 0.8, SO_3 and H_2SO_4 concentrations displayed similar distinct peaks, but the individual concentration changes of H_2SO_4

and SO_3 were less than the 5×10^5 or the 3×10^5 molecules cm^{-3} thresholds to be classified as an event. An example of this occurs on September 29th-30th, 2023 and is shown in Figure S3(B).

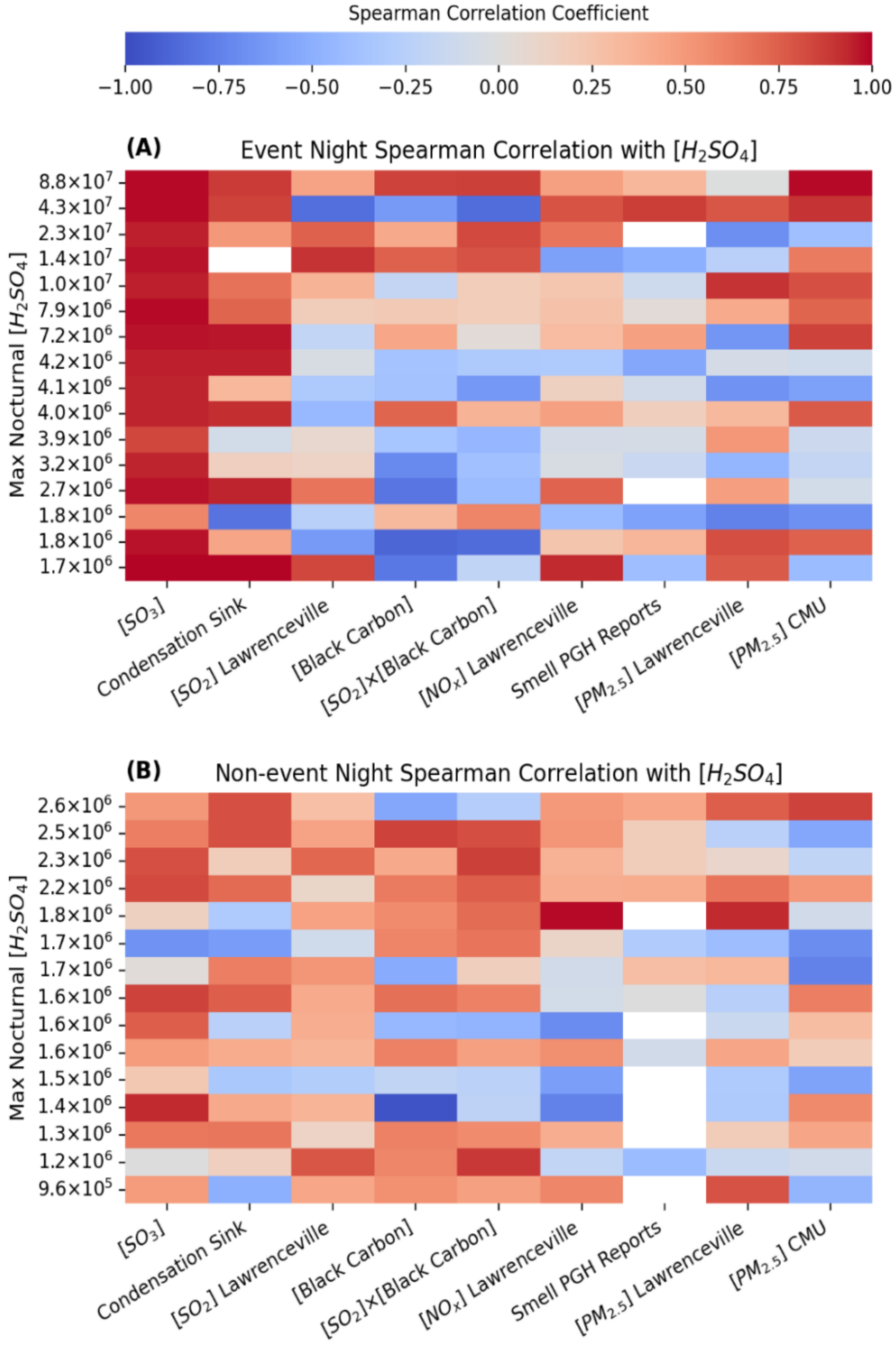

Figure S4. Spearman correlation of H_2SO_4 concentration with measurements taken during the Pittsburgh campaigns during (A) event nights and (B) non-event nights. Rows are sorted by the maximum nightly H_2SO_4 concentration. White spaces either had no measurements available or all measurements were zero.

The correlations of H_2SO_4 and SO_3 with SmellPGH and H_2S are heavily dependent on the trajectory of the plume. For example, the observed increases in H_2S concentration at the ACHD North Braddock and Liberty sites did not always correlate with the H_2SO_4 events due to the location of the sensors. In addition, not every H_2SO_4 formation event correlates with increased SmellPGH reports; Figure S4 shows no correlation or

negative correlation for 12 event nights. Nights with no correlation may be due to inconsistent reporting by residents during the nighttime when people are sleeping.

5. H_2SO_4 24-hour timeline

Daytime H_2SO_4 was observed during all campaigns, as shown in Figure S5. Higher daytime concentrations were correlated with ultraviolet radiation and low condensation sinks. Daytime H_2SO_4 events had fewer SmellPGH reports and lower SO_3 concentrations than nighttime events, as shown in Figure S5. The lower SO_3 concentrations are likely a result of faster conversion to H_2SO_4 due to higher water dimer concentrations.[2] The reduced number of SmellPGH reports may be a result of changes in meteorological conditions, such as boundary layer expansion, and/or lower concentrations of H_2S.

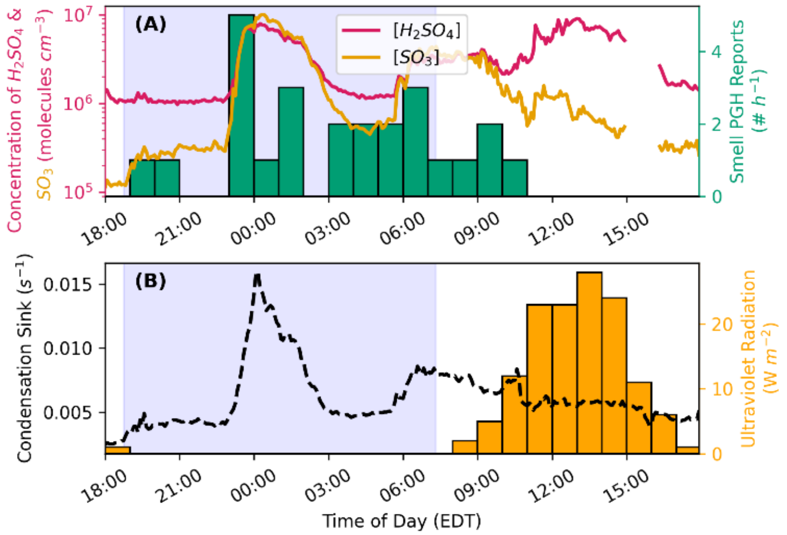

Figure S5. 24-hour timeline of (A) H_2SO_4 and SO_3 concentrations and SmellPGH reports and (B) condensation sink and ultraviolet radiation from October 11th at 18:00 to October 12th at 18:00 EDT, 2023.

Figure S6 depicts the 1-3 nm size distribution measured by the Condensation Particle Counters for Atmospheric Rapid Measurements (CPC FARM)[9] with the corresponding H_2SO_4, SO_3, HSO_4^-•H_2SO_4, and CS on 10/11/23 to 10/12/23. The high nighttime sulfuric acid resulted in the appearance of clusters and particles in the smallest CPC FARM bin of 1.6 nm. This could also be due to the CPC FARM detecting sulfuric acid.[10] The sulfuric acid dimer concentration HSO_4^-•H_2SO_4, as measured by the CIMS at 195 m/z, exhibited almost no increase. This contrasts with the daytime H_2SO_4 to dimer trend observed during the daytime. The high nocturnal H_2SO_4 did not result in the formation of dimers or nucleation of particles due to the high condensation sink of 0.016 s^{-1} during peak H_2SO_4. The daytime CS during peak H_2SO_4 and dimer concentration was only 0.006 s^{-1}.

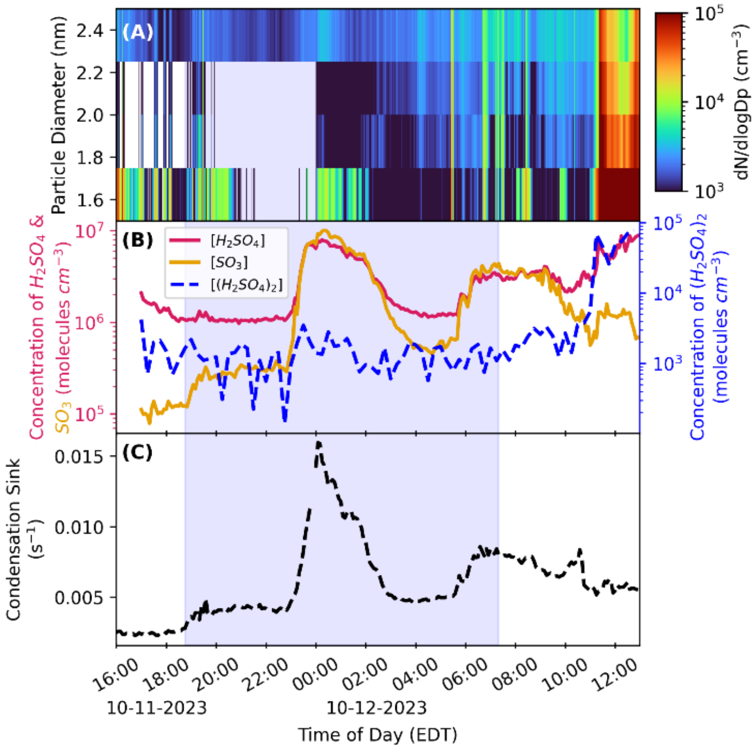

Figure S6 Observations for 10/11/23 to 10/12/23 measured by the (A) CPC FARM for the 1-3 nm particle size distribution, (B) CIMS for the H_2SO_4 (red), SO_3 (yellow), and HSO_4^-•H_2SO_4 (dashed blue), and (C) the condensation sink (CS) from the particle size distribution.

6. Plume Pittsburgh Gaussian Plume Models of Mon Valley Works Emissions

The Gaussian plume model created by Plume Pittsburgh shows potential emissions from the Mon Valley Works blowing towards the measurement sites on many nights with H_2SO_4 events. Figure S7 shows the Gaussian plume model on October 11th and 12th, 2023, three different times with recent SmellPGH reports shown as triangles. Between 22:00 and 23:00 EDT on October 11th, 2023, the plume shifts direction towards Pittsburgh. At midnight on October 12th, 2023, the concentrated portion of the plume is shown to travel over CMU, but it shifts direction towards the northeast before reaching Lawrenceville.

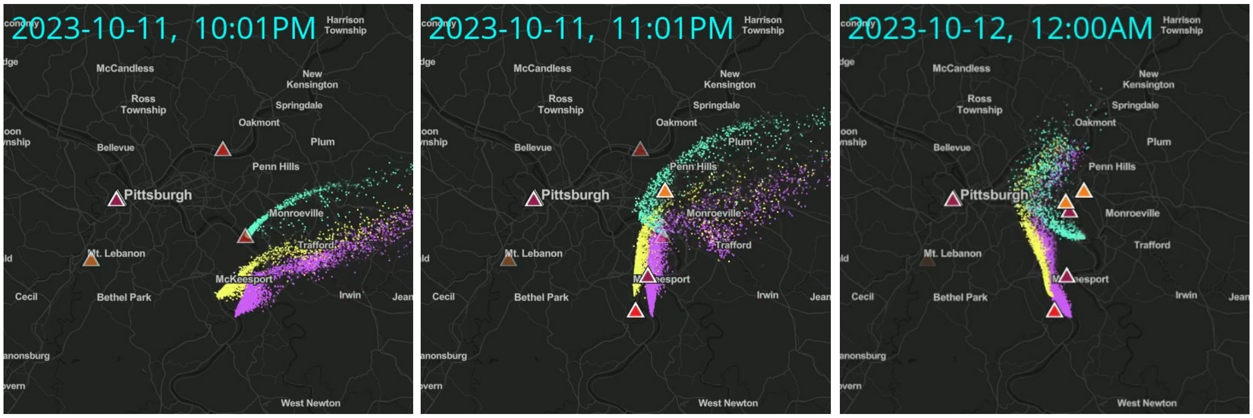

Figure S7. Gaussian plume model of potential emissions from the three plants in the Mon Valley Works complex from Plume PGH for the night of October 11th, 2023, to October 12th, 2023. Recent SmellPGH reports are marked by a triangle. The color of the triangles represents the reported intensity of the smell, with maroon as the most intense and yellow as the least intense.

Figure S8 shows a Gaussian plume model from October 12th, 2024. Like Figure S7, Figure S8 shows the plume's direction shifting towards Pittsburgh during the night as a result of wind direction changes. Unlike in Figure S7(C), Figure S8(C) shows the plume covering both CMU and Lawrenceville, suggesting that both sites measured the plume at roughly the same time. Both the PurpleAir measurements and Plume Pittsburgh model show that the air masses containing H_2SO_4 and increased particle concentrations originate from the southeast of Pittsburgh and likely contain emissions from the Mon Valley Works steel plant, coke plant, or steel processing plant. Additionally, when the airmass changes direction at ~23:00 EDT, there are new SmellPGH reports overlapping with the modeled plume.

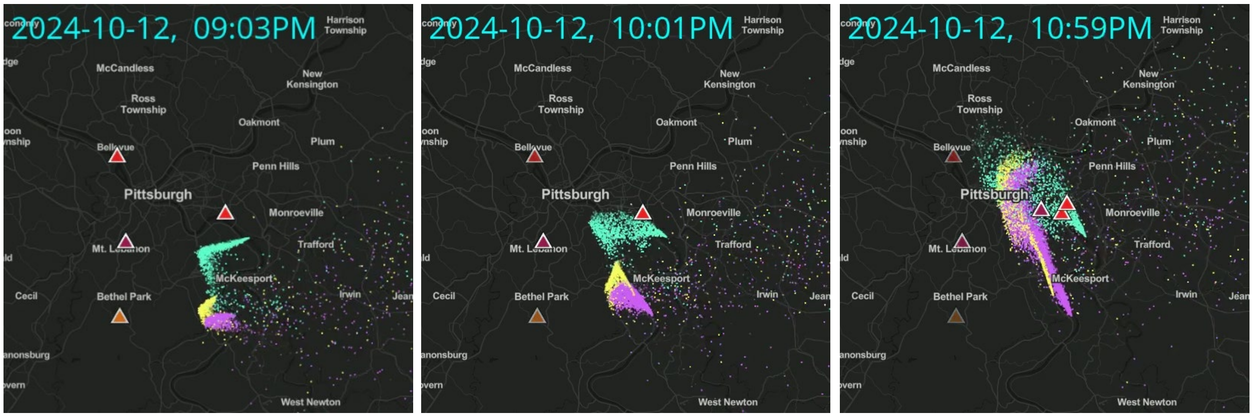

Figure S8. Gaussian plume model of potential emissions from the Mon Valley Works complex from Plume Pittsburgh for the night of October 12th, 2024. Recent SmellPGH reports are marked by a triangle. The color of the triangles represents the reported intensity of the smell, with maroon as the most intense and yellow as the least intense.

7. Xact $PM_{2.5}$ Metal Composition

Previous studies show that metal oxides, which are emitted from coal combustion and steel milling, can oxidize both H_2S and SO_2.[11–16] Although metal oxides containing only one metal have not been shown to oxidize sulfur to SO_3 at atmospheric conditions, a ternary metal oxide containing Fe, Mn, and Ze was observed to form sulfate at room temperature.[17] Additionally, the mass fraction of Fe, Mn, and Zn, see Figure 4 and Figure S9, correlated with the second event on October 12th, 2023 and the event on October 12th, 2024.

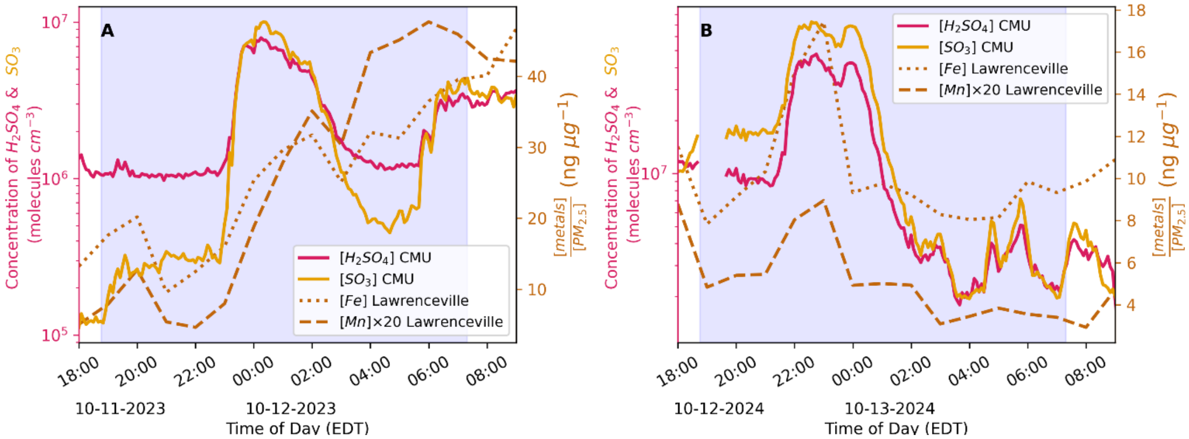

Figure S9. Measurements of concentration of H_2SO_4 and SO_3 at CMU and mass fraction of Fe and Mn at Lawrenceville on October 11th–12th, 2023 (A) and October 12th–13th, 2024 (B).

Figure S10 shows the Spearman correlation between H_2SO_4 and mass fraction of trace metals relative to total $PM_{2.5}$ mass concentration, measured by an Xact 625i and Thermo RP Partisol-Plus 2025 in Lawrenceville. The measurements in Figure S10 were filtered to include metals with a larger correlation with H_2SO_4 and excluded measurements with metal concentrations below the minimum detection limit. The mass fraction of trace metals in coal combustion emissions changes depending on coal composition, making it difficult to identify a tracer element. Previous aerosol particles emitted from combustion of different origins of coal frequently observed Se, Mn, Zn, Al, Ti, and Fe in the emissions.[18–21] Emissions from the production of coke include many of the same metals, but previous studies have also observed Ni.[21,22]

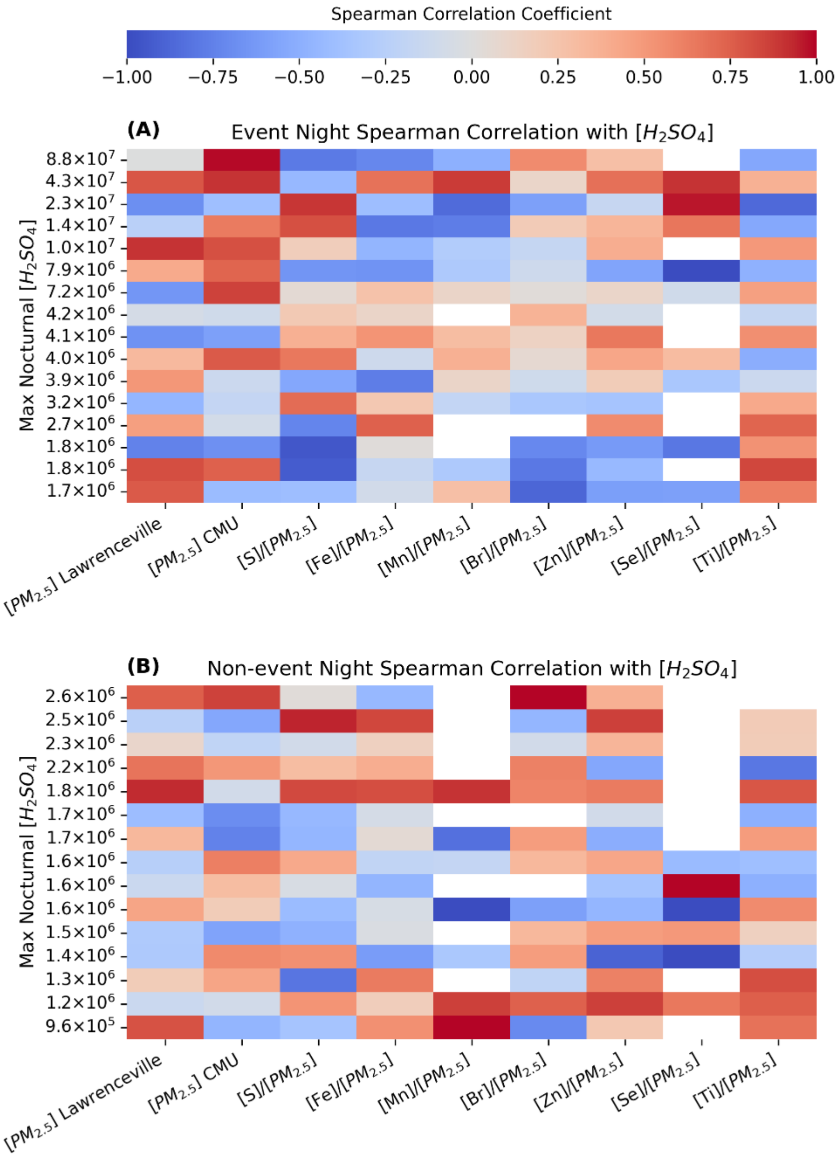

Figure S10. Spearman correlation coefficients for event nights (A) and non-event nights (B) between H_2SO_4, $PM_{2.5}$, and mass fractions of trace metals measured in Lawrenceville

8. CIMS m/z ratios correlated with H_2SO_4

Peak correlation was calculated using the zscore function in the SciPy package in Python. All listed peaks had correlation scores greater than 0.5. Once correlated peaks were identified, the Tofware software package was used to identify potential compositions based on m/z location and isotope ratios. Three peaks identified by zscore had m/z ratios of 93.00, 108.99, and 140.98, which had potential chemical formulae of $HNO \cdot NO_3^-$, $HONO \cdot NO_3^-$, and $HNO_4 \cdot NO_3^-$, respectively. These are not the only possible structural guesses from the formulae, and it is not possible to definitively determine the compound identity using CIMS. Figure S11 shows the correlation between H_2SO_4, the three identified peaks, and NO_x concentrations measured in Lawrenceville. Nights with higher correlation between H_2SO_4 and NO_x generally had higher correlation with the three identified peaks. Reactive nitrogen species may contribute to the formation of H_2SO_4 as previous measurements have shown nitrate enhances the oxidation of SO_2 on Fe_2O_3. [23]

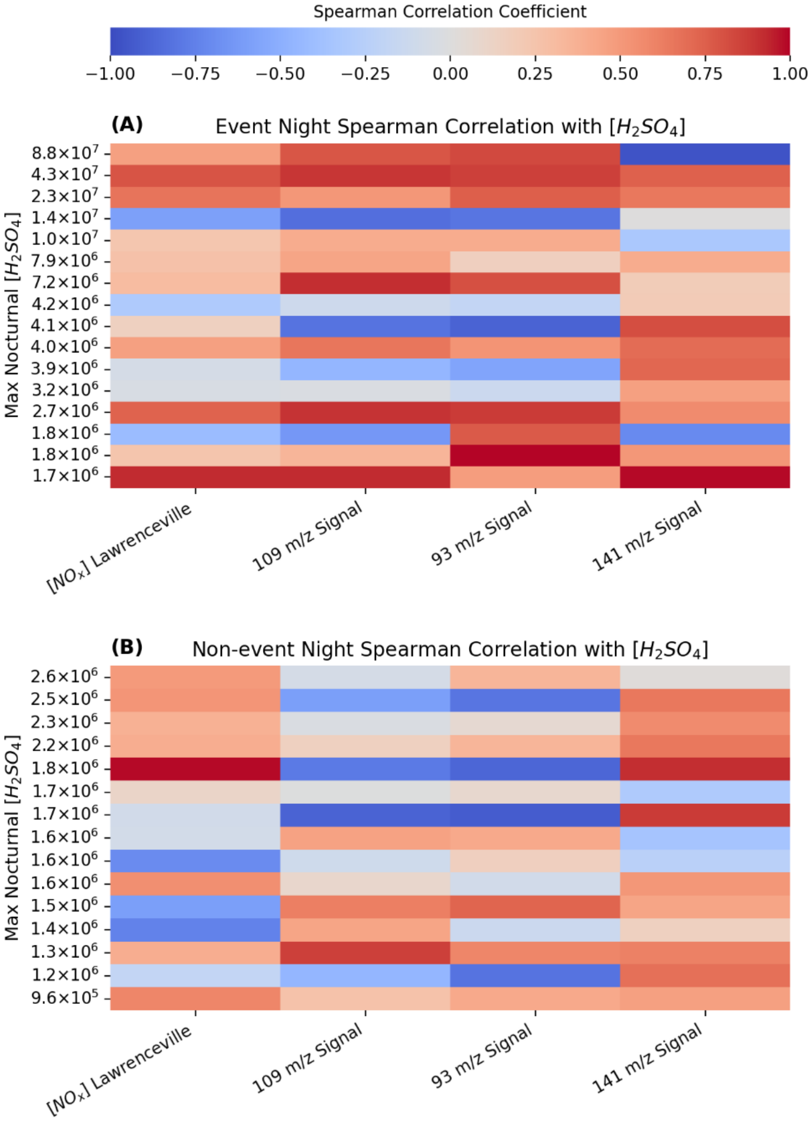

Figure S11. Correlations for event (A) and non-event nights (B) between H_2SO_4 and NO_x, 109 m/z, 93 m/z, and 141 m/z, which have possible compositions of $HNO \cdot NO_3$-, $HONO \cdot NO_3$-, and $HNO_4 \cdot NO_3$-.

References

(1) *Google Maps*. Google Maps. https://www.google.com/maps (accessed 2025-08-27).
(2) Yao, L.; Fan, X.; Yan, C.; Kurtén, T.; Daellenbach, K. R.; Li, C.; Wang, Y.; Guo, Y.; Dada, L.; Rissanen, M. P.; Cai, J.; Tham, Y. J.; Zha, Q.; Zhang, S.; Du, W.; Yu, M.; Zheng, F.; Zhou, Y.; Kontkanen, J.; Chan, T.; Shen, J.; Kujansuu, J. T.; Kangasluoma, J.; Jiang, J.; Wang, L.; Worsnop, D. R.; Petäjä, T.; Kerminen, V.-M.; Liu, Y.; Chu, B.; He, H.; Kulmala, M.; Bianchi, F. Unprecedented Ambient Sulfur Trioxide (SO3) Detection: Possible Formation Mechanism and Atmospheric Implications. *Environ. Sci. Technol. Lett.* **2020**, *7* (11), 809–818. https://doi.org/10.1021/acs.estlett.0c00615.
(3) Fomete, S. K. W.; Johnson, J. S.; Myllys, N.; Jen, C. N. Experimental and Theoretical Study on the Enhancement of Alkanolamines on Sulfuric Acid Nucleation. *J. Phys. Chem. A* **2022**, *126* (25), 4057–4067. https://doi.org/10.1021/acs.jpca.2c01672.
(4) Heinritzi, M.; Simon, M.; Steiner, G.; Wagner, A. C.; Kürten, A.; Hansel, A.; Curtius, J. Characterization of the Mass-Dependent Transmission Efficiency of a CIMS. *Atmos. Meas. Tech.* **2016**, *9* (4), 1449–1460. https://doi.org/10.5194/amt-9-1449-2016.
(5) Fomete, S. K. W.; Johnson, J. S.; Myllys, N.; Neefjes, I.; Reischl, B.; Jen, C. N. Ion–Molecule Rate Constants for Reactions of Sulfuric Acid with Acetate and Nitrate Ions. *J. Phys. Chem. A* **2022**, *126* (44), 8240–8248. https://doi.org/10.1021/acs.jpca.2c02072.
(6) Arnold, S. T.; Morris, R. A.; Viggiano, A. A.; Jayne, J. T. Ion Chemistry Relevant for Chemical Ionization Detection of SO3. *Journal of Geophysical Research: Atmospheres* **1995**, *100* (D7), 14141–14146. https://doi.org/10.1029/95JD01004.
(7) Viggiano, A. A.; Perry, R. A.; Albritton, D. L.; Ferguson, E. E.; Fehsenfeld, F. C. STRATOSPHERIC NEGATIVE-ION REACTION RATES WITH H2SO4. *J. Geophys. Res.* **1982**, *87*, 7340–7342. https://doi.org/10.1029/JC087iC09p07340.
(8) Dwivedi, P.; Matz, L. M.; Atkinson, D. A.; Herbert H. Hill, J. Electrospray Ionization-Ion Mobility Spectrometry: A Rapid Analytical Method for Aqueous Nitrate and Nitrite Analysis. *Analyst* **2004**, *129* (2), 139–144. https://doi.org/10.1039/B311098B.
(9) Cheng, D.; Amanatidis, S.; Lewis, G. S.; Jen, C. N. Fast and Sensitive Measurements of Sub-3 Nm Particles Using Condensation Particle Counters For Atmospheric Rapid Measurements (CPC FARM). *Atmospheric Measurement Techniques* **2025**, *18* (1), 197–210. https://doi.org/10.5194/amt-18-197-2025.
(10) Cheng, D.; Kiguru ,Joy; and Jen, C. N. Detection Efficiency of a Water Condensation Particle Counter Using Electrically Neutral Sulfuric Acid and Sulfuric Acid-Dimethylamine Clusters. *Aerosol Science and Technology, Aerosol Research Letters* **2025**, *59* (8), 915–920. https://doi.org/10.1080/02786826.2025.2496486.
(11) Lowell, P. S.; Schwitzgebel, K.; Parsons, T. B.; Sladek, K. J. Selection of Metal Oxides for Removing SO_2 From Flue Gas. *Ind. Eng. Chem. Proc. Des. Dev.* **1971**, *10* (3), 384–390. https://doi.org/10.1021/i260039a018.
(12) Lapina, O. B.; Bal'zhinimaev, B. S.; Boghosian, S.; Eriksen, K. M.; Fehrmann, R. Progress on the Mechanistic Understanding of SO2 Oxidation Catalysts. *Catalysis Today* **1999**, *51* (3), 469–479. https://doi.org/10.1016/S0920-5861(99)00034-6.
(13) Fang, H.; Zhao, J.; Fang, Y.; Huang, J.; Wang, Y. Selective Oxidation of Hydrogen Sulfide to Sulfur over Activated Carbon-Supported Metal Oxides. *Fuel* **2013**, *108*, 143–148. https://doi.org/10.1016/j.fuel.2011.05.030.
(14) Yang, Q.; Liu, G.; Falandysz, J.; Yang, L.; Zhao, C.; Chen, C.; Sun, Y.; Zheng, M.; Jiang, G. Atmospheric Emissions of Particulate Matter-Bound Heavy Metals from Industrial Sources. *Science of The Total Environment* **2024**, *947*, 174467. https://doi.org/10.1016/j.scitotenv.2024.174467.
(15) Davydov, A. A.; Marshneva, V. I.; Shepotko, M. L. Metal Oxides in Hydrogen Sulfide Oxidation by Oxygen and Sulfur Dioxide: I. The Comparison Study of the Catalytic Activity. Mechanism of the

Interactions between H2S and SO2 on Some Oxides. *Applied Catalysis A: General* **2003**, *244* (1), 93–100. https://doi.org/10.1016/S0926-860X(02)00573-2.
(16) Chughtai, A. R.; Brooks, M. E.; Smith, D. M. Effect of Metal Oxides and Black Carbon (Soot) on SO2/O2/H2O Reaction Systems. *Aerosol Science and Technology* **1993**, *19* (2), 121–132. https://doi.org/10.1080/02786829308959626.
(17) Gupta, N. K.; Kim, E. J.; Baek, S.; Bae, J.; Kim, K. S. Ternary Metal Oxide Nanocomposite for Room Temperature H2S and SO2 Gas Removal in Wet Conditions. *Sci Rep* **2022**, *12* (1), 15387. https://doi.org/10.1038/s41598-022-19800-6.
(18) Amdur, M. O.; Sarofim, A. F.; Neville, Matthew.; Quann, R. J.; McCarthy, J. F.; Elliott, J. F.; Lam, H. Fuan.; Rogers, A. E.; Conner, M. W. Coal Combustion Aerosols and Sulfur Dioxide: An Interdisciplinary Analysis. *Environ. Sci. Technol.* **1986**, *20* (2), 138–145. https://doi.org/10.1021/es00144a004.
(19) Linak, W. P.; Yoo, J.-I.; Wasson, S. J.; Zhu, W.; Wendt, J. O. L.; Huggins, F. E.; Chen, Y.; Shah, N.; Huffman, G. P.; Gilmour, M. I. Ultrafine Ash Aerosols from Coal Combustion: Characterization and Health Effects. *Proceedings of the Combustion Institute* **2007**, *31* (2), 1929–1937. https://doi.org/10.1016/j.proci.2006.08.086.
(20) Sambandam, B.; Devasena, T.; Islam, V. I. H.; Prakhya, B. M. Characterization of Coal Fly Ash Nanoparticles and Their Induced in Vitro Cellular Toxicity and Oxidative DNA Damage in Different Cell Lines. *Indian J Exp Biol* **2015**, *53* (9), 585–593.
(21) Weitkamp, E. A.; Lipsky, E. M.; Pancras, P. J.; Ondov, J. M.; Polidori, A.; Turpin, B. J.; Robinson, A. L. Fine Particle Emission Profile for a Large Coke Production Facility Based on Highly Time-Resolved Fence Line Measurements. *Atmospheric Environment* **2005**, *39* (36), 6719–6733. https://doi.org/10.1016/j.atmosenv.2005.06.028.
(22) Mu, L.; Peng, L.; Liu, X.; Bai, H.; Song, C.; Wang, Y.; Li, Z. Emission Characteristics of Heavy Metals and Their Behavior During Coking Processes. *Environ. Sci. Technol.* **2012**, *46* (11), 6425–6430. https://doi.org/10.1021/es300754p.
(23) Kong, L. D.; Zhao, X.; Sun, Z. Y.; Yang, Y. W.; Fu, H. B.; Zhang, S. C.; Cheng, T. T.; Yang, X.; Wang, L.; Chen, J. M. The Effects of Nitrate on the Heterogeneous Uptake of Sulfur Dioxide on Hematite. *Atmospheric Chemistry and Physics* **2014**, *14* (17), 9451–9467. https://doi.org/10.5194/acp-14-9451-2014.
